# Radiation-induced Cavernous Malformation as a Late Sequelae of Stereotactic Radiosurgery for Epilepsy

**DOI:** 10.7759/cureus.2308

**Published:** 2018-03-11

**Authors:** Ethan A Winkler, Caleb Rutledge, Mariann Ward, Tarik Tihan, Patricia K Sneed, Nicholas Barbaro, Paul Garcia, Michael McDermott, Edward F Chang

**Affiliations:** 1 Department of Neurological Surgery, University of California, San Francisco; 2 Neuropathology, University of California, San Francisco; 3 Department of Radiation Oncology, University of California, San Francisco; 4 Department of Neurological Surgery, University of Indiana; 5 Neurology, University of California, San Francisco; 6 Department of Neurological Surgery, University of California San Francisco

**Keywords:** radiosurgery, gamma knife, epilepsy, cavernous malformation, mesial temporal sclerosis

## Abstract

Stereotactic radiosurgery (SRS) is a promising treatment for medically intractable mesial temporal lobe epilepsy. SRS for epilepsy has had an acceptable safety profile with reports of radiation-induced vascular malformations confined to central nervous system pathologies with prominent angiogenesis – namely, primary brain tumors, metastases, and arteriovenous malformations. Theoretical risks for radiation-induced lesions following radiosurgery for epilepsy have yet to be established. Of 13 patients treated in a pilot trial for medial temporal lobe epilepsy, one developed multiple delayed radiation-induced cavernous malformations following radiosurgery. This patient received a prescription dose of 20 Gy delivered to the amygdala, anterior hippocampus, and parahippocampal gyrus. Eight years following treatment, computed tomography imaging demonstrated an evolving hyperdensity in the mesial temporal lobe. Magnetic resonance imaging confirmed multiple T2 hypointense lesions with a mixed-signal intensity core in the left parahippocampal gyrus and anterior temporal lobe. The patient was initially managed conservatively. However, recurrent hemorrhage ultimately caused an acute deterioration in mental status, aphasia, and hemiparesis, necessitating surgical resection. Pathology confirmed radiation-induced cavernous malformations. This represents the first case of a radiation-induced vascular lesion as a long-term sequela of radiosurgery for epilepsy and illustrates the potential for this complication even when low doses are used in patients without angiogenic lesions. Optimal timing and indications for surgical resection of radiation-induced cavernous malformations prior to the development of neurologic symptoms warrant further refinement. Long-term vigilance and clinical monitoring are required.

## Introduction

Radiosurgery is a less invasive treatment option for medically intractable mesial temporal lobe epilepsy (MTLE) for those not eligible or wanting an open surgical procedure, such as anterior temporal lobectomy. In prospectively enrolled trials, Gamma Knife® radiosurgery (Elekta, Stockholm, Sweden) for MTLE has had an acceptable safety profile [1-2]. It is the subject of a multicenter phase three randomized controlled trial comparing its efficacy with open surgery – the Radiosurgery or Open Surgery for Epilepsy (ROSE) Trial (NCT00860145). However, the radiosurgical treatment of MTLE is not without risk, including post-procedure headaches, visual field deficits, verbal memory impairment, radiation necrosis, delayed cyst formation, and steroid-dependent cerebral edema. With long-term follow-up, hypothetical risks for radiation-induced tumors or vascular malformations have yet to be established.

Cerebral cavernous malformations (CMs) are angiographically occult vascular lesions comprised of dilated vascular channels with thin walls and no intervening brain parenchyma [3]. Although classically ascribed to sporadic or familial autosomal dominant etiologies, cranial radiotherapy has become an increasingly recognized causative factor for the de novo formation of cerebral CMs. The majority of published cases of radiation-induced CMs, however, involve radiotherapy during childhood [4-5]. Far fewer published cases exist on radiation-induced cavernous malformations as a sequela of adult radiosurgery, and these have focused on cerebral pathologies with prominent angiogenesis, e.g., primary central nervous system (CNS) tumors, brain metastases, and/or arteriovenous malformations [3]. Here, we offer the first report of multiple radiation-induced CMs culminating in symptomatic hemorrhage as long-term sequelae of Gamma Knife radiosurgery for epilepsy.

## Case presentation

Initial history and presentation

**Figure 1:**
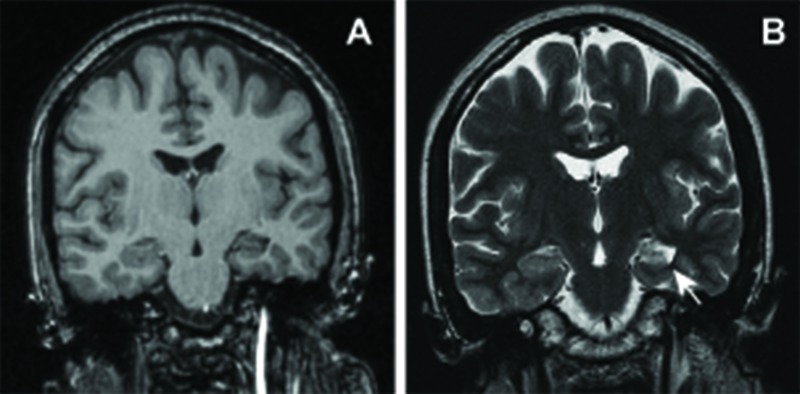
Preoperative MRI Coronal non-contrast T1- weighted (A) and T2-weighted (B) images demonstrating assymetric volume loss and hyperintensity of the left hippocampus consistent with mesial temporal sclerosis. Arrows, region of interest.

A 29-year-old right-handed woman presented with medically refractory epilepsy in December of 1997. Her seizures had initially begun at the age of four years old and were characterized by the absence of an aura, facial automatisms (e.g., lip smacking/biting), and tendency to progress to secondarily generalized tonic-clonic seizures. Her seizures occurred roughly 20 times a month with a nocturnal predominance and remained refractory to multiple anti-epileptic drug trials, including carbamazepine, sodium valproate, and phenobarbital. Her neurologic examination revealed no deficits. However, a more detailed neuropsychological workup demonstrated impaired verbal memory. As part of her workup, an interictal electroencephalogram (EEG) demonstrated high amplitude left temporal spikes and magnetic resonance imaging (MRI) demonstrated asymmetric volume loss of the left hippocampus with subtle T2-hyperintensity consistent with mesial temporal sclerosis without evidence of additional lesions (Figure 1).

Radiosurgery and interim clinical course

**Figure 2:**
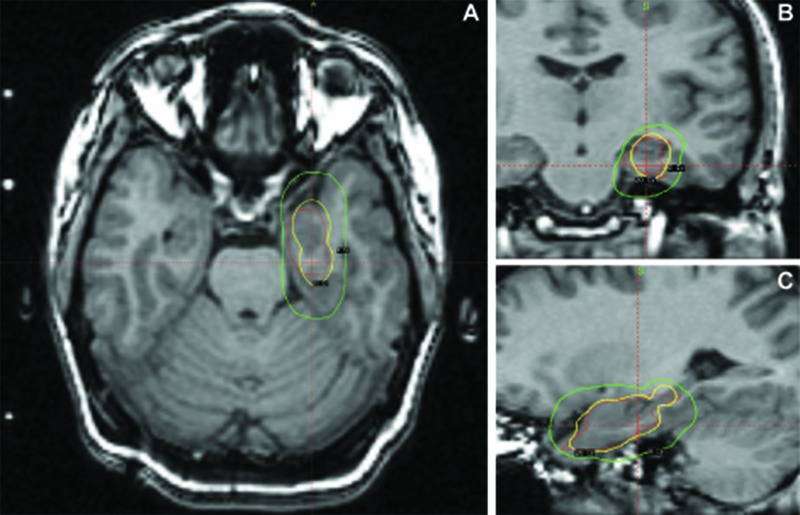
Preprocedural MRI for radiosurgical planning with superimposed dosimetry data. Axial (A), coronal (B) and sagittal (C) non-contrast T1-weighted images obtained for radiation planning. Eighty percent of the target volume (red) was covered by the 20 Gy isodose surface (yellow). The peripheral target volume covered by the 8 Gy isodose surface was also shown (green).

Initial attempts to improve seizure control through further refinement of anti-epileptic medications, including the addition of topiramate, were unsuccessful. By 2001, her seizure frequency remained unchanged. Based on the available data, she was offered a left anterior temporal lobectomy. However, as a result of religious and cultural norms prohibitive of shaving off her hair, she elected to proceed with a less invasive procedure. She was enrolled in the National Institutes of Health (NIH)-sponsored Gamma Knife Radiosurgery for Temporal Lobe Epilepsy Pilot Trial and randomized to receive 20 Gy treatment – this was in lieu of a 24 Gy dose provided to the other randomized treatment group. In July 2001, she was treated with Gamma Knife radiosurgery applied to a 1.7 x 2.8 x 1.9 cm region encompassing the left mesial temporal lobe, including the amygdala, anterior hippocampus, and parahippocampal gyrus. A prescription dose of 20 Gy was delivered to the 50% isodense contour, which covered 80% of the target region, given strict brainstem, optic chiasm, and optic nerve dose constraints (Figure 2). The procedure was uncomplicated and the patient was discharged the same day.

The patient initially had a good result to treatment and achieved seizure freedom within one-year post-procedure. Within three years of her radiosurgery, however, her seizures returned and she was experiencing complex partial seizures every one to two weeks. The semiology of these seizures was similar to the prior events, except that they often led to worsening of her mild, baseline psychosis. Over the ensuing years, her neuropsychiatric health also began to decline, including disorganization of thought, ideas of reference, and paranoia. Attempts to further localize her seizure focus was untaken to evaluate whether she would still be a candidate for surgical resection. In May 2008, a non-contrast MRI demonstrated evolving encephalomalacia with surrounding gliosis and decreasing fluid-attenuated inversion recovery (FLAIR) hyperintensity of the left anterior mesial temporal lobe structures, including the amygdala, anterior hippocampus, and parahippocampal gyrus, consistent with evolving changes related to her Gamma Knife radiosurgery. These imaging findings subsequently stabilized on repeat imaging in April 2009. The following month, she was hospitalized for continuous video-EEG monitoring, which confirmed left temporal lobe sharp waves. Her seizures, however, now arose independently from both left and right temporal regions, and after multidisciplinary discussion, it was deemed that she was not a surgical candidate nor would she benefit from intracranial recordings. As a result, continued efforts to optimize anti-epileptic medications were undertaken with limited and often transient benefit, including the addition of levetiracetam, lamotrigine, pregabalin, and gabapentin, and continuation of her carbamazepine therapy.

Detection of radiation-induced cavernous malformation

**Figure 3:**
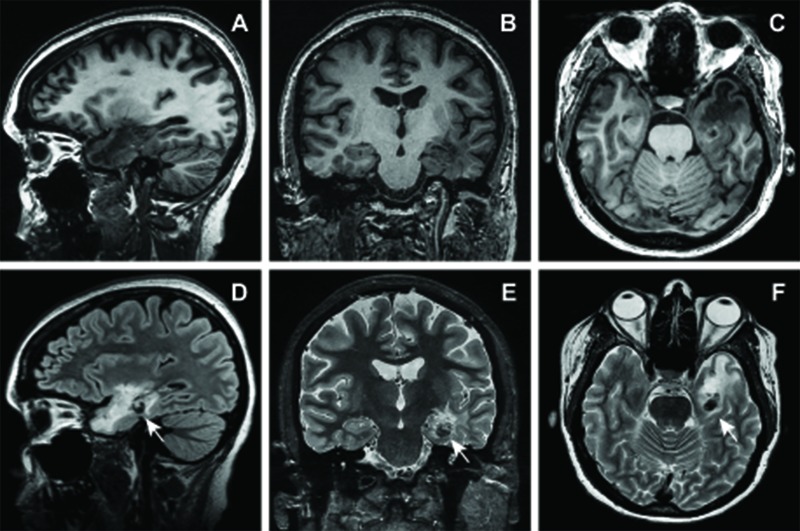
MRI obtained 9.5 years following radiosurgery showing suspected radiation-induced lesion. Sagittal, coronal and axial T1-weighted without contrast (A-C) and T2-weighted (D-F) images demonstrating interval development of a multilobular hetereogenous lesion within the left parahippocampal gyrus which is T2 hypointense with sublte intrinsic T1 shortening with associated susceptibility. A second small focus with similar imaging characteristics is also seen within the left anterior temporal lobe. Arrows, region of interest.

From November 2009 to December 2010, her complex partial seizures resulted in multiple falls from standing which led to emergency department visits for mild head trauma. Non-contrast computed tomography (CT) scans of her head over this interval demonstrated stable hypodensity within the left anterior mesial temporal lobe consistent with known radiation-induced encephalomalacia. Within the region of the parahippocampal gyrus, however, there was a new hyperdense focus concerning for either acute hemorrhage or calcification, which was subsequently demonstrated to expand on subsequent scans. In January 2011, these findings prompted a non-contrast MRI of the brain, which demonstrated a heterogeneous 1.3 x 1.0 cm multi-lobulated T2 hypointense lesion with mixed-signal intensity core in the left parahippocampal gyrus. A second 4.0 x 5.0 mm lesion was also observed within the anterior temporal lobe white matter with subtle T1 shortening, a T2 hyperintense center, and a surrounding T2 hypointense rim with an associated susceptibility-related signal loss (Figure 3). The radiographic appearance was consistent with radiation-induced cavernous malformations, but the differential also consisted of radiation-induced tumors or hemorrhage secondary to radionecrosis.

Re-presentation and intracerebral hemorrhage

From January 2011 to February 2016, she continued to have persistent complex partial seizures requiring further optimization of her anti-epileptic regimen, including trials of zonisamide and clobazam, as well as a further decline in mental health. In February 2016, the patient was brought to an outside emergency department by the family who noted a two-week decline in the level of interaction and lethargy. On exam, she was awake and alert but demonstrated limited interaction and was perseverative on formal language evaluation. She displayed no other focal neurologic findings. A non-contrast CT demonstrated a 3.9 x 3.0 x 3.1 cm hyperdense lesion in the left mesial temporal lobe with significant peri-lesional edema and mass effect, causing roughly 1 cm of left-to-right midline shift. A non-contrast MRI again demonstrated a heterogenous multilobulated lesion within the left parahippocampal gyrus with a mixed signal intensity core, surrounding T2-hypointense rim, and marked susceptibility. She was subsequently hospitalized for seven days and discharged with a decadron taper.

**Figure 4:**
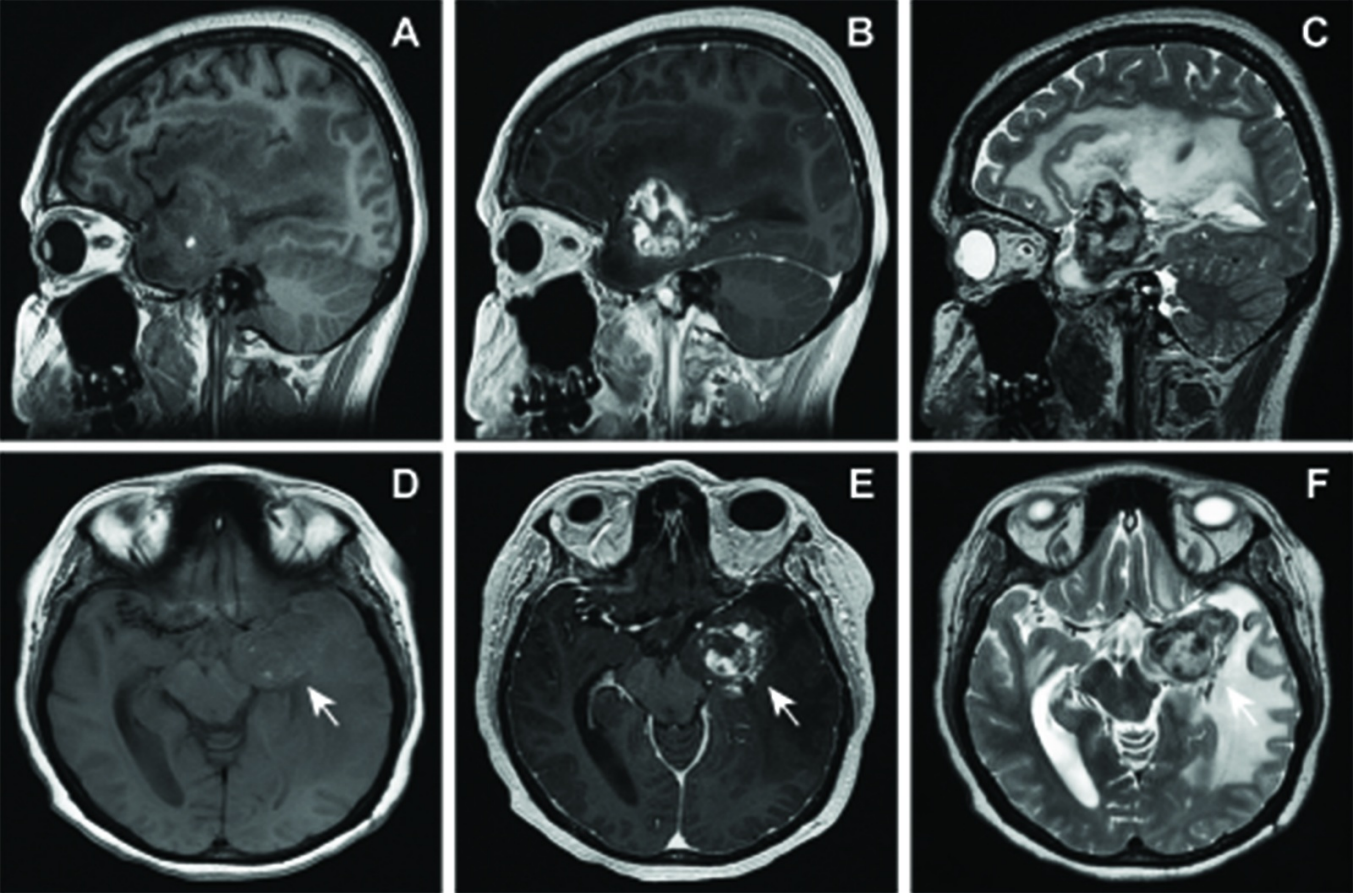
Preoperative MRI obtained 15.5 years following radiosurgery showing interval expansion and hemorrhage of radiation-induced lesion. Sagittal and axial T1 weighted without constrast (A,D), with gadolinium contrast (B, E), and T2-weighted (C, F) images demonstrating progressive enlargement of lesion within left parahippocampal gyrus with small amount of intrinsic T1 signal, peripheral enhancement and extensive surrounding edema. The lesion exerts extensive mass effect with effacement of the left lateral ventricle, 7 mm of midline shift and herniation of the left uncus leading to compression of the left cerebral peduncle and midbrain. Arrows, region of interest.

Three days following her discharge, her husband witnessed her fall from standing from an apparent loss of consciousness, which resulted in her striking her head and having a subsequent generalized tonic-clonic seizure. On examination, she was awake and alert but had a new right superior quadrantanopia and elements of a receptive aphasia on examination, including dysnomia and numerous paraphasic errors with conversation. A non-contrast CT scan of the head re-demonstrated a 4.0 x 3.2 x 3.1 cm hyperdense lesion in the left mesial temporal lobe with increased density, but similar mass effect causing nearly 1.0 cm of left-to-right midline shift with herniation of the left uncus. A subsequent MRI with and without contrast demonstrated a peripherally enhancing heterogeneous mass arising within the left parahippocampal gyrus with marked susceptibility, low blood flow on arterial spin labeling, and prominent peri-lesional edema (Figure 4). The patient was admitted to the intensive care unit for further observation.

Surgical procedure

The next morning, the patient became progressively lethargic and developed a right-sided hemiparesis with worsening aphasia. She was taken urgently to the operating room for a left frontotemporal craniotomy for evacuation of the hematoma, resection of suspected cavernous malformation, and an anterior temporal lobectomy. Excision of the anterior temporal lobe was accomplished with subpial dissection, and the hemorrhagic lesion was identified and excised en bloc for pathologic examination. The parenchyma was noted to be more gliotic and firm than normal. The case was uncomplicated, and the patient awoke with no new neurologic deficits and was transferred back to the intensive care unit.

Histopathologic findings

The en bloc surgical specimen from the temporal lobe contained a hemorrhagic, firm, and rubbery mass with a rim of surrounding brain parenchyma measuring 3.5 x 3.2 x 1.5 cm in three dimensions. The hemorrhagic mass had specks of yellow discoloration and had a centrally cystic component and adjacent vascular tissue.

**Figure 5:**
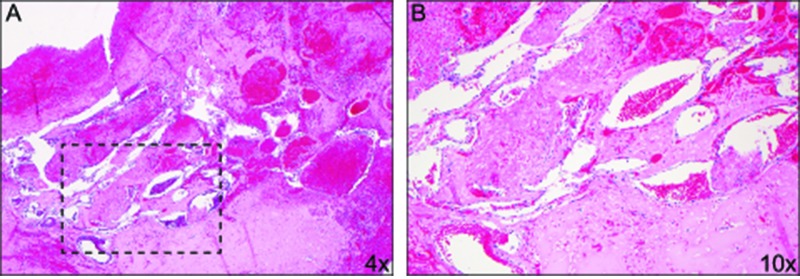
Histopathology Low-magnification (A) and higher magnification (B) of hemotoxylin & eosin staining of intraoperative specimen showing a vascular lesion comprised of compact array of markedly hyalinized vessels without intervening neural tissue, extensive mural thickening without elastic lamina and marked extravasation of erythrocytes consitent with a radiation-induced cavernous malformation. The adjacent brain parenchyma was hemosiderin laden with moderate to marked gliosis, macrophage infiltration, white matter vacuolation, and neuronal loss. The adjacent microvasculature were thick walled with mural necrosis consistent with radiation effects.

The microscopic evaluation demonstrated a vascular lesion consistent with a CM composed of a compact array of markedly hyalinized vessels without an intervening stroma and extravasation of erythrocytes. Almost all the vascular structures within the lesion demonstrated extensive mural thickening without elastic lamina. The adjacent brain parenchyma also demonstrated moderate to marked gliosis, white matter vacuolation, neuronal loss in gray matter, hemosiderin deposition, and scattered macrophages, as well as scattered thick-walled capillaries and small venules, some of which had mural necrosis; this was consistent with radiation effect (Figure 5).

Postoperative course

**Figure 6:**
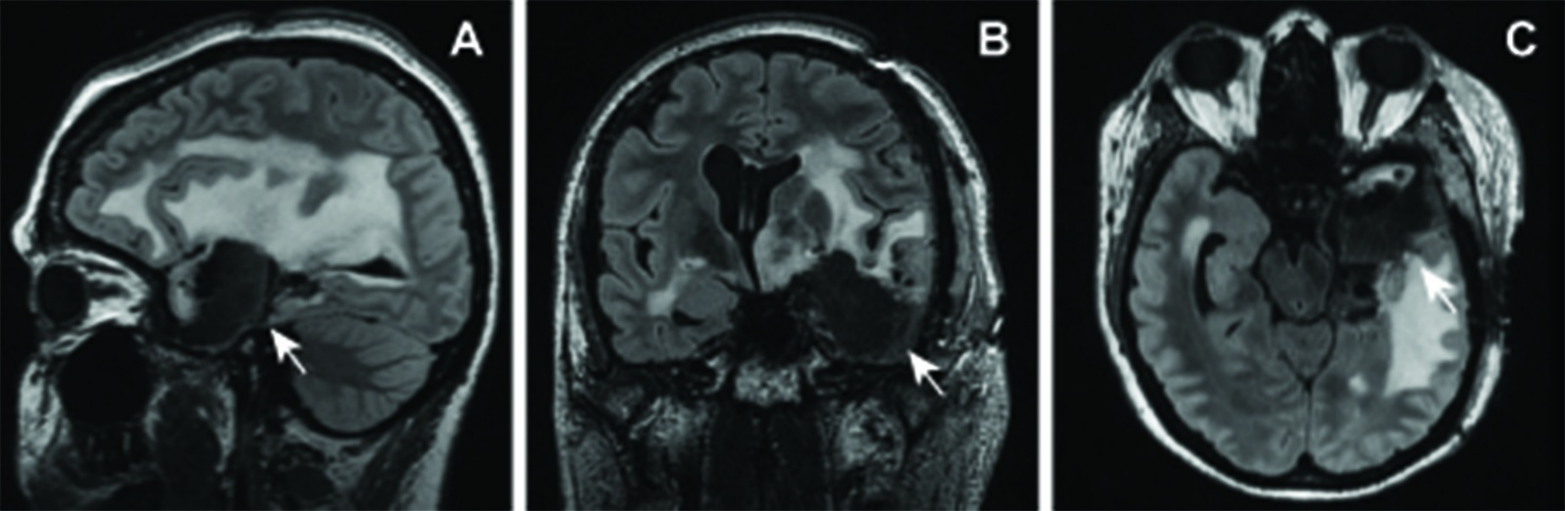
Postoperative MRI Sagittal (A), coronal (B), and axial (C) FLAIR images demonstrating interval left frontotemporal craniotomy with gross total resection of the cavernous malformation and anterior temporal lobectomy with expected postoperative changes. Arrows, region of interest.

Her postoperative course was notable for a persistent, but gradually improving aphasia. However, her right hemiparesis gradually improved. Postoperative MRI demonstrated a gross total resection of the hemorrhagic lesion (Figure 6). She remained seizure-free for the duration of her hospital stay and reported no further seizures at three months of follow-up.

## Discussion

We report a case of radiation-induced cavernous malformations in the parahippocampal gyrus and anterior temporal lobe following stereotactic radiosurgery for MTLE in a patient with mesial temporal sclerosis. The cavernous malformation ultimately hemorrhaged repeatedly, resulting in worsening aphasia, a right hemiparesis, and alteration in mental status, requiring surgical resection. Prior pathologic examination of temporal lobe structures following radiosurgery for epilepsy have only demonstrated degrees of radiation effect, including perivascular inflammation, vascular sclerosis, necrosis, edema, a proliferation of microglia, and neuronal loss [6]. To date, this report represents the first description of a cavernous malformation as a sequela of radiosurgery for epilepsy, whereas prior reports of radiation-induced cavernous malformations have been confined to pathologies with prominent angiogenesis, e.g. brain neoplasia, metastasis, or arteriovenous malformations [3]. Therefore, this report suggests that irradiated brain, even without persistent pathologic angiogenic stimulation, is at risk for the formation of a CM following stereotactic radiosurgery.

In the general population, the prevalence of cerebral cavernous malformations is estimated to be between 0.3 to 0.6% [7]. Although expected to be higher in those with prior cranial irradiation, the incidence or radiation-induced cerebral cavernous malformations is largely unknown [4]. Following childhood radiotherapy for brain tumors, the cumulative incidence of CMs has been estimated to be 3 - 3.9 % and 5 - 14 % at 10 and 15 years following radiotherapy, respectively [4-5]. However, the incidence of radiation-induced CMs has yet to be reported in adults and/or with non-neoplastic conditions but is suggested to vary as a function of cumulative radiation dose. Several authors have reported that radiation-induced CMs are typically observed with fractionated radiotherapy greater than 30 Gy [3-5]. Others have reported that radiation-induced CMs may still arise with lower radiation doses with fractionated therapy (20-24 Gy) or with single fraction radiosurgery with a prescription dose between 20 to 24 Gy [8]. The presented case, which utilized single fraction radiosurgery of 20 Gy for epilepsy, further supports this point.

The presented case also highlights several additional attributes of radiation-induced cavernous malformations that have been previously reported. With cranial radiation for brain neoplasia, a median latency from the time of cranial radiotherapy to the detection of radiation-induced cavernous malformations ranges from eight to 12 years [3-4]. Consistent with these reports, the first radiographic evidence suggestive of a cavernous malformation was detected eight years following SRS in the presented case. This lesion was subsequently demonstrated to expand and was definitely demonstrated on an MRI 14 months later. As with an estimated 41 to 63% of radiation-induced cavernous malformations [3-5], this was not a solitary lesion but rather demonstrated at least one additional satellite lesion within the irradiated field in the anterior temporal lobe white matter. Additionally, the histopathologic appearance of the radiation-induced lesion was indistinguishable from sporadic CMs, as previously reported [3].

Effective guidelines for treatment are presently lacking. Hemorrhage – the most feared consequence of cerebral CMs – has been detected in an estimated 40-56% of radiation-induced cavernous malformations [3, 8]. In many instances, the hemorrhage is asymptomatic and only detected radiographically, as initially seen in the present report. Early reports suggested a higher rate of hemorrhage with radiation-induced CMs when compared to sporadic CMs [8-9]. More recently, this has been debated [3-4], and a recent report has suggested that the annual hemorrhage rate is 4.2% per person/year for radiation-induced CMs, which was not significantly different from that observed in sporadic CMs [3]. A prior bleed in radiation-induced CMs and sporadic CMs greatly increases the risk for re-hemorrhage, a value which declines over the ensuing five years [3, 7, 10]. Female gender also portends a greater risk of hemorrhage in some, but not all, studies in sporadic CMs [7]. Therefore, the presented patient had multiple risk factors for hemorrhage, and when ultimately symptomatic, surgical resection was required.

## Conclusions

This represents the first case of a radiation-induced vascular lesion as a long-term sequela of radiosurgery for epilepsy and illustrates the potential for this complication, even when low doses are used in patients without angiogenic lesions. Optimal timing and indications for surgical resection of radiation-induced cavernous malformations prior to the development of neurologic symptoms warrant further refinement. Long-term vigilance and clinical monitoring are required.
